# A geospatial approach to understanding inequalities in accessibility to primary care among vulnerable populations

**DOI:** 10.1371/journal.pone.0210113

**Published:** 2019-01-07

**Authors:** Jason A. Gilliland, Tayyab I. Shah, Andrew Clark, Shannon Sibbald, Jamie A. Seabrook

**Affiliations:** 1 Department of Geography, Western University, London, ON, Canada; 2 Human Environments Analysis Laboratory, Western University, London, ON, Canada; 3 School of Health Studies, Schulich School of Medicine and Dentistry, Western University, London, ON, Canada; 4 Department of Paediatrics, Western University, London, ON, Canada; 5 Department of Epidemiology and Biostatistics, Western University, London, ON, Canada; 6 Children’s Health Research Institute, London, ON, Canada; 7 Lawson Health Research Institute, London, ON, Canada; 8 Department of Family Medicine, Western University, London, ON, Canada; 9 School of Food and Nutritional Sciences, Brescia University College, London, ON, Canada; Newcastle University, UNITED KINGDOM

## Abstract

Many Canadians experience unequal access to primary care services, despite living in a country with a universal health care system. Health inequalities affect all Canadians but have a much stronger impact on the health of vulnerable populations. Health inequalities are preventable differences in the health status or distribution of health resources as experienced by vulnerable populations. A geospatial approach was applied to examine how closely the distribution of primary care providers (PCPs) in London, Ontario meet the needs of vulnerable populations, including people with low income status, seniors, lone parents, and linguistic minorities. Using enhanced two step floating catchment area (E2SFCA) method, an index of geographic access scores for all PCPs and PCPs speaking French, Arabic, and Spanish were separately developed at the dissemination area (DA) level. To analyze how PCPs are distributed, comparative analyses were performed in association with specific vulnerable groups. Geographical accessibility to all PCPs, and PCPs who speak specific minority languages vary considerably across the city of London. Access scores for French- and Arabic-speaking PCPs are found comparatively high (mean = 2.85 and 1.01 respectively) as compared to Spanish-speaking PCPs (mean = 0.47). Additionally, many areas with high proportions of vulnerable populations experience low accessibility. Despite its exploratory nature, this study offers insight into intra-urban distributions of geographical accessibility to primary care resources for vulnerable groups. These findings can facilitate health researchers and policymakers in the development of recommendations to increase levels of accessibility of specific population groups in underserved areas.

## Introduction

Primary health care (PHC) is acknowledged as a foundation of any high performing health care system that provides access to health care services for all health-related needs and problems, providing person-focused care over time in a continuous and coordinated fashion [1]. Across Canadian provinces, PHC models include a wide range of provider groups and clinicians, each with their own funding and accountability relationships [1, 2]. Among these PHC practitioners/professionals, family physicians and nurse practitioners provide a full range of primary care services, and are the gatekeepers who facilitate easier access to many other health care services [3, 4]. This includes prescription medication, referrals to specialized care, and diagnostic imaging services [3].

Despite having a universal health care system, health care inequality in Canada is a multidimensional challenge that varies by geographic location and across different vulnerable populations. Over the past decade, Canadian provinces and territories have made considerable progress implementing PHC reform strategies to improve access and quality of health services; however, despite these efforts many Canadians continue to experience unequal access to health care [5–7]. This is an increasingly prevalent issue because of recent demographic shifts in Canada, including an aging population, growing income inequality, changing immigration patterns, and increasing urbanization of the population. On the Island of Montreal, for example, Paez et al. (2010) found large disparities in accessibility to primary care facilities between seniors and non-seniors [8]. Other research has shown that people who do not speak the language of majority are much more likely to experience difficulties with the health care system [9, 10]. A recent study of French-speaking immigrants in a predominantly English-speaking Canadian city identified how a lack of available health care services in French can lead to dissatisfaction with care, emotional stress, and/or delay in seeking out health care [9]. Language is often a barrier for immigrants and refugees, with some patients only able to communicate with health care providers through an interpreter [11–14]. Those without access to an interpreter may experience errors in translation or in understanding the implications of their health status [12, 15, 16].

To address inequalities in PHC provision in a demographically diverse population, an integrative and multidisciplinary approach is needed. The rapid advancement of methods in geographic information science have changed the way that human geography is involved in health services research [17]. We posit that GIS-based methods of spatial analysis can be used for examining inequalities in accessibility to primary care among vulnerable populations.

This research uses novel geographic methods to examine the spatial distribution of family physicians, general practitioners, and nurse practitioners (from here on referred to as “primary care providers” or “PCPs”) in a mid-sized Canadian city (London, Ontario). This study hypothesized that geographical accessibility to PCPs, and more specifically, PCPs who speak specific languages (other than English), will vary considerably across a metropolitan area. Furthermore, we submit that areas having poor access to PCPs will be even more disadvantaged for vulnerable populations. The specific objectives of this study are: 1) to examine the inequalities in geographical accessibility to PCPs among vulnerable populations; and 2) to understand how closely PCPs speaking specific languages in a mid-sized Canadian City meet the needs of three minority language speaking populations: French, Arabic, and Spanish.

## Methods

We conducted a population-based study examining the geographical accessibility to all PCPs inclusively, as well as French-, Arabic-, and Spanish-speaking PCPs exclusively, within the city of London, Ontario. This study is part of a comprehensive project conducted with the South West Local Health Integration Network (SWLHIN) to inform the development of recommendations that support equitable access to primary care for individuals within the region.

### Data sources

PCP Information were provided by the College of Physicians and Surgeons of Ontario in July 2015. This data included the name of the PCP, office address, and languages spoken of all PCPs that reside within the Southwestern Ontario region encompassing the city of London. This data were verified by staff of HealthForceOntario, a governmental agency responsible for planning, recruitment, retention, transition and distribution of health practitioners in Ontario [18]. As an outcome of this verification process, a comprehensive list of primary care providers [19] that have a comprehensive primary care practice (i.e., have a roster of patients enrolled) were prepared for analysis. In other words, family physicians that work in non-primary care settings (i.e., hospital, focused practice, etc.) were not considered for computing geographic accessibility.

Population data were obtained from the 2011 Census of Canada and 2011 National Household Survey [20]. The following population data were used in this study:

- Population by dissemination block (DB);
- Total number of people per dissemination area (DA) whose Mother Tongue (first language) is (a) French, (b) Arabic, or (c) Spanish;
- Number of people per DA over the age of 65;
- Number of census families headed by a lone parent; and
- Census families who live below Statistics Canada’s low-income measure.

The vulnerable population and language speaking population groups are measured at the DB and DA levels, respectively. The DA is the smallest geographic unit for which statistics Canada releases comprehensive census data, whereas DB, an area bounded on all sides by roads and/or other geographic boundaries (e.g., rivers), is the smallest geographic area for which population and dwelling counts are disseminated [London DAs (n = 570), population size (mean: 642, SD: 389); London DBs (n = 2516), population size (mean = 160, SD:222)].

### Setting

**Fig 1:**
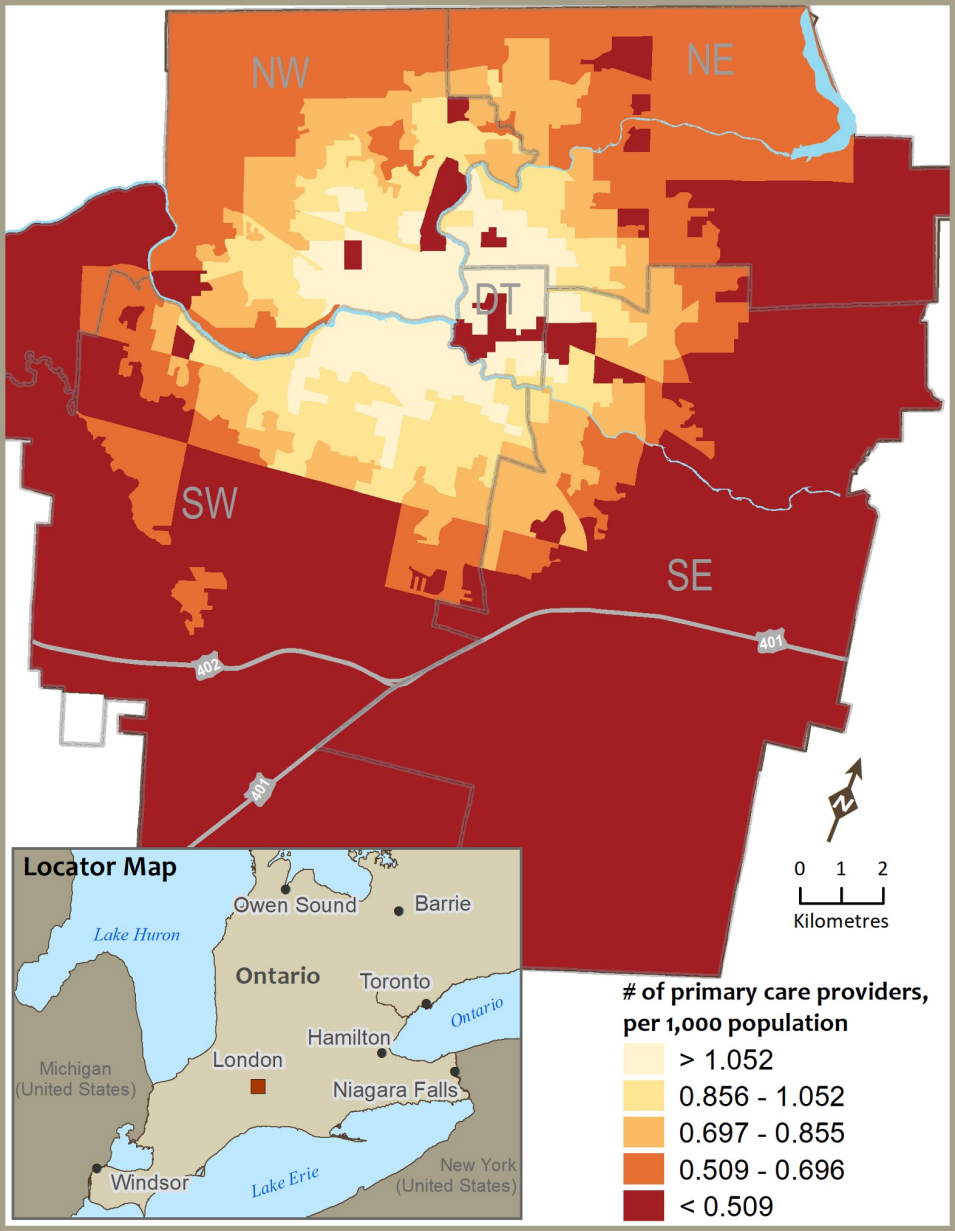
Geographic accessibility to primary care providers (PCPs) within 15 minutes driving distance across the City of London, Ontario.

The overarching project was conducted for the entire SWLHIN region, which includes five sub-LHIN areas with a total population of nearly 1 million. We chose to focus on London for this study on language barriers as it is the region’s largest city (366,151 inhabitants in 2011) and it is also ethnically diverse, as more than 1 out of five (21%) inhabitants were born outside of Canada. The city of London is the largest urban settlement in Southwestern Ontario. It is located along the highway 401, the busiest road in North America, approximately halfway between Toronto, Ontario and Detroit, Michigan (see Fig 1, Locator Map). For reference, we show how the City of London is divided into five large geographic areas (i.e., Central London and Downtown, North East, North West, South East, and South West) [21].

### Data analysis

The mean and standard deviation was used to describe continuous variables, and percentages were used for categorical outcomes. The Pearson correlation coefficient assessed the strength and direction of the association between continuous variables. Language-specific accessibility scores were compared using a Kruskal-Wallis H test. A p value <0.05 was considered statistically significant. All statistical analyses were performed in SPSS (IBM SPSS Statistics for Windows, version 24, IBM Corp., Armonk, NY). The geographical accessibility to PCPs (and PCPs speaking specific languages) was measured using the E2SFCA method. The original 2-step floating catchment area (2SFCA) method was developed by Radke and Mu [22] and later modified by Luo and Wang [23]. It is a sophisticated technique for measuring supply-to-demand ratios within certain catchment areas, and is implemented in GIS using the following two steps:

Step 1: For each physician location j, search all population locations (k) that are within a threshold drive time (d_0_) from location j (i.e., catchment area j), and compute the physician-to-population ratio R_j_ within the catchment area:

$$R_{j}=\frac{S_{j}}{\sum_{k\in \{d_{kj}\leq d_{0}\}}P_{k}},$$

where P_k_ is the population of DB k whose centroid falls within the catchment (i.e., d_kj_< = d_0_), S_j_ is the number of physicians at location j, and d_kj_ is the drive time between k and j.

Step 2: For each population location i, search all physician locations (j) that are within the threshold drive time (d_0_) from location i (i.e., catchment area i), and sum up the R_j_ at these locations:

$$A_{i}^{F}=\sum_{j\in \{d_{ij}\leq d_{0}\}}R_{j}=\sum_{j\in \{d_{ij}\leq d_{0}\}}\left(\frac{S_{j}}{\sum_{k\in \{d_{ij}\leq d_{0}\}}P_{k}}\right)$$

where $A_{i}^{F}$ represents the accessibility at DB i based on the two-step FCA method, R_j_ is the physician-to-population ratio at physician location j that falls within the catchment area at i (i.e., d_ij_< = d_0_), and d_ij_ is the drive time between i and j.

The E2SFCA used for this study is based on a modified 2SFCA method that introduced distance decay concept within global catchment area [24, 25]. This enhancement requires that the demand population (Step 1) and the service provision-to-population ratios (Step 2) are multiplied by a weighting W_kj_ based on a function of the distance-decay between the demand population located at k and service supply location at j:

$$R_{j}=\frac{S_{j}}{\sum_{k\in \{d_{kj}\leq d_{max}\}}P_{k}∙W_{kj}}$$

$$A_{k}=\sum_{j\in \{d_{kj}\leq d_{max}\}}R_{j}∙W_{kj}$$

An ArcGIS add-in tool developed by Langford, Higgs and Fry [24] was used to calculate the E2SFCA accessibility scores for all PCPs and PCPs speaking French, Arabic, and Spanish. The following distance decay parameters were used in all measures: a Gaussian decay function with the bandwidth of 50, 15-minute drive time (as a cut-offs distance) to define the catchment areas, geocoded locations for PCP practice sites, and DB/DA centroids. The selection of 15-minute drive time is based on the premise that catchment area should be equal to the average commute time during office hours that individuals living in one of five geographic areas may travel to seek care in the neighbouring geographic area. While no data are available on private automobile ownership among different groups or neighbourhoods within London, according to 2016 Labour Force Survey, of those who commuted to work in the City of London, private vehicle (i.e., car, truck or van as a driver/passenger) was by far the most commonly used mode of transportation (i.e., 82.6% versus public transit (9.2%) and walk, bicycle and all other modes (8.1%)) which are comparatively higher than provincial figures (77.9%) [26]. Whereas the percentage of commuters who used public transit (9.2%) is on the lower side (14.6% in Ontario) [26]. We used geographic area (DB/DA) centroid points to represent where people live (and would be traveling from) for computing geographic accessibility for all PCPs and PCPs speaking specific languages, respectively. To perform comparative analysis with vulnerable groups, geographic accessibility scores for all PCPs were collapsed into a binary arrangement (below and above)–where city average was used as a cut-off to distinguish between the poor (below) and good (above) geographical accessibility areas (i.e., 0.73 PCPs-per-1000 population). Similarly, three variables (low-income, senior population, and lone-parent families) were dichotomized into below and above city average (i.e., 16.9%, 14.5%, 18.9%, respectively) as shown in Table 1. In case of access scores for PCPs speaking specific language, a geospatial mapping approach was used to analyze the spatial distribution patterns of geographic accessibility where a manually defined single scale classification was applied.

**Table 1:** Descriptive statistics of geographic accessibility scores for PCPs (all PCPs, specific language-speaking PCPs) along with vulnerable populations in London, Ontario.

Variables (DA level: 570)	Mean	SD	Min	Max	N
Access score for all PCPs	0.77	0.30	0.00	1.39	570
Access score for French speaking PCPs	2.85	1.04	0.54	4.69	495
Access score for Arabic speaking PCPs	1.01	0.36	0.19	1.73	350
Access score for Spanish speaking PCPs	0.47	0.23	0.03	0.91	421
% of Low income families	15.71	13.94	0.00	68.40	557
% of Seniors (65+) population	14.52	8.80	0.00	57.14	570
% of Lone parent families	18.78	9.92	0.00	56.76	570

## Results

### Descriptive statistics

In the City of London, there were 267 active PCPs (as of 2015), the majority of whom work in office-based primary care practices. There was, however, no indication as to the number of patients rostered and their FTE equivalent. The city of London has a variety of PCPs that practice primary care in languages other than English, with 13 of them practicing in French, seven in Arabic, and four in Spanish. An examination of the first languages of the population indicates that London is linguistically diverse with only 77.8% of the population having English as its first language or ‘mother tongue’, 1.3% of the population have French as first language, and 19.2% do not have either official language (English or French) as their mother tongue. The most frequently reported non-official languages spoken as primary languages are Spanish (2.6%) and Arabic (2.4%). The summary of the geographic accessibility (E2SFCA access scores) for PCPs along with the vulnerable populations is provided in Table 1.

### Geographic accessibility for all PCPs and distribution of vulnerable populations

**Fig 2:**
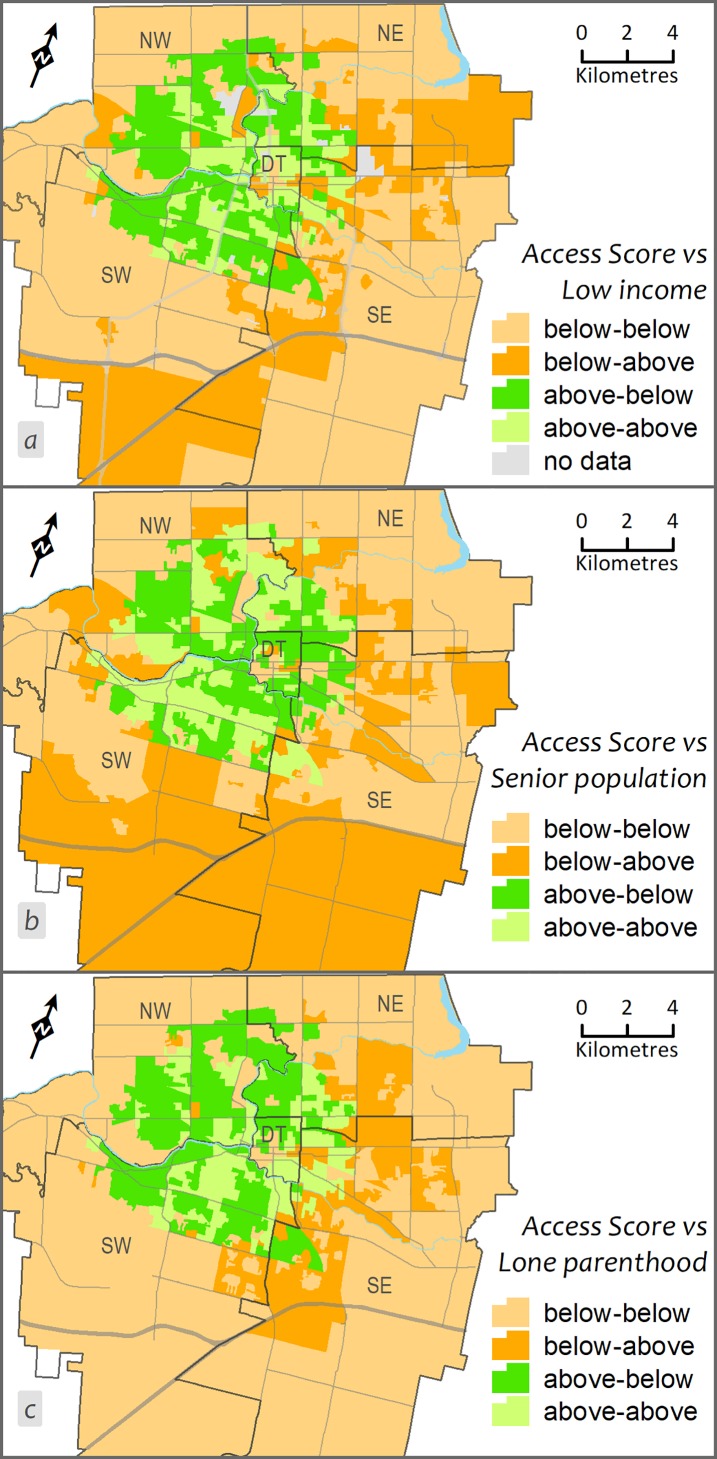
**Cross-classification between geographic accessibility score for PCPs and a) prevalence of low income families, b) the percentage of the population aged 65 years and older (seniors), c) percentage of lone parent families.** Below and above indicates the values below and above the cut-off values determined for each variable separately. DT = Downtown/central, NW = Northwest, NE = Northeast, SW = Southwest, and SE = Southeast.

Fig 1 provides an illustration of how DA geographic accessibility scores for all PCPs that were determined using ESFCA within a 15-minute drive distance vary across the city. To demonstrate intra-urban variation, we categorized the access scores into five classes using quintile classification where the last two classes (<0.509; 0.509–0.696) indicates low accessibility areas (i.e., DAs). Access scores for all PCPs and percent of vulnerable groups were compared using correlation analysis (Pearson’s r) and cross-classification matrix on two categorical variables (crosstab mapping). The positive but weak correlations between the access score and low-income (r = 0.14; p = 0.001) and senior’s population (r = 0.12; p = 0.01), and a nonsignificant correlation between access score and lone-parenthood (r = 0.05; p = 0.22) indicated the distribution of PCPs is associated, albeit weakly, with the distribution of vulnerable populations. Fig 2 presents a spatial cross-classification between the binary form of two variables—access scores for all PCPs and vulnerable groups. A set of four classes based on the below and above categories from two variables were used to cross-classify the geographic units (i.e., DA): below category for access score versus below category from the vulnerable group (for example, say ‘% of low-income families’); similarly, below-above, above-below, and above-above. The key combination where below category from access score and above category from vulnerable populations (below-above) in this cross-classified map represented the dissimilarity or mismatch in the distribution of PCPs in relation to health care needs (see Fig 2).

### Geographic accessibility to minority language speaking populations

**Fig 3:**
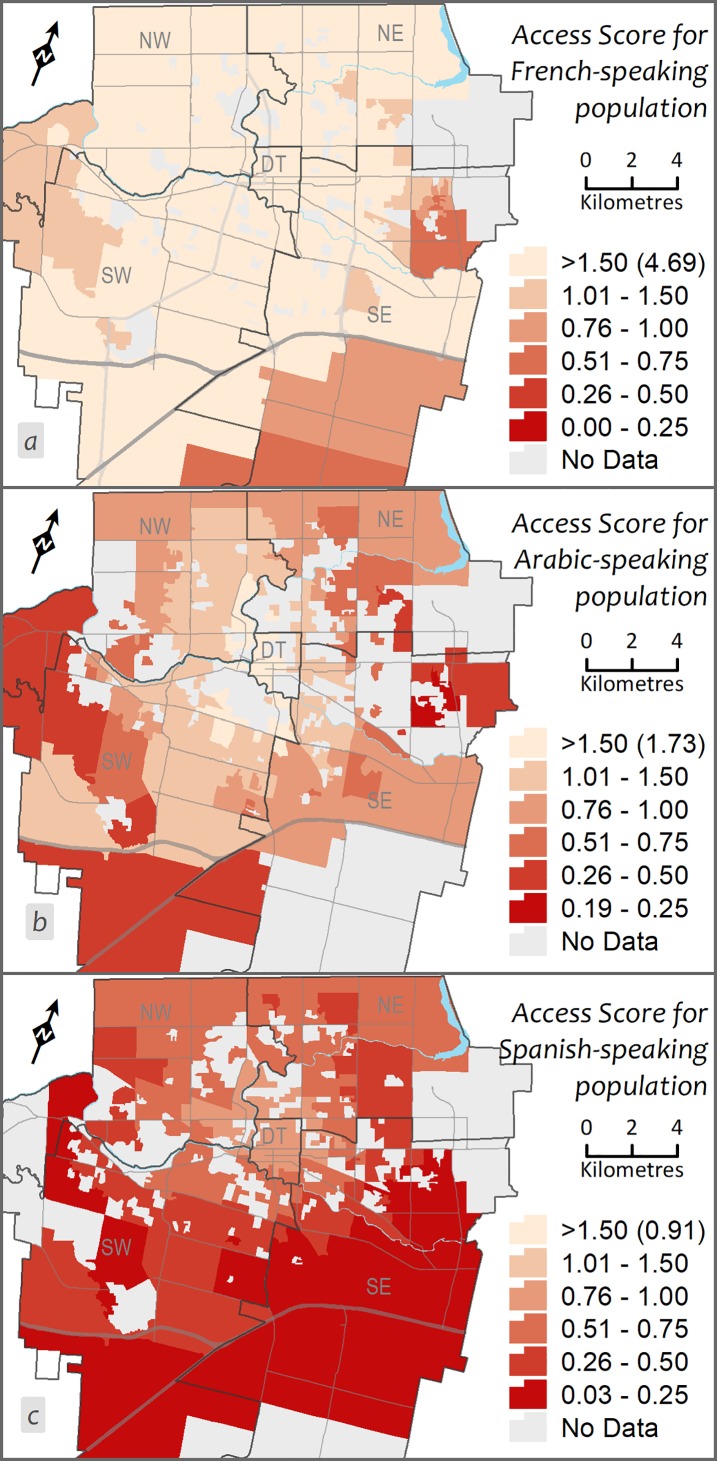
Geographic accessibility to primary care providers (PCPs) within 15 minutes driving distance by: a) French-speaking, b) Arabic-speaking, and c) Spanish-speaking PCPs language groups.

**Fig 4:**
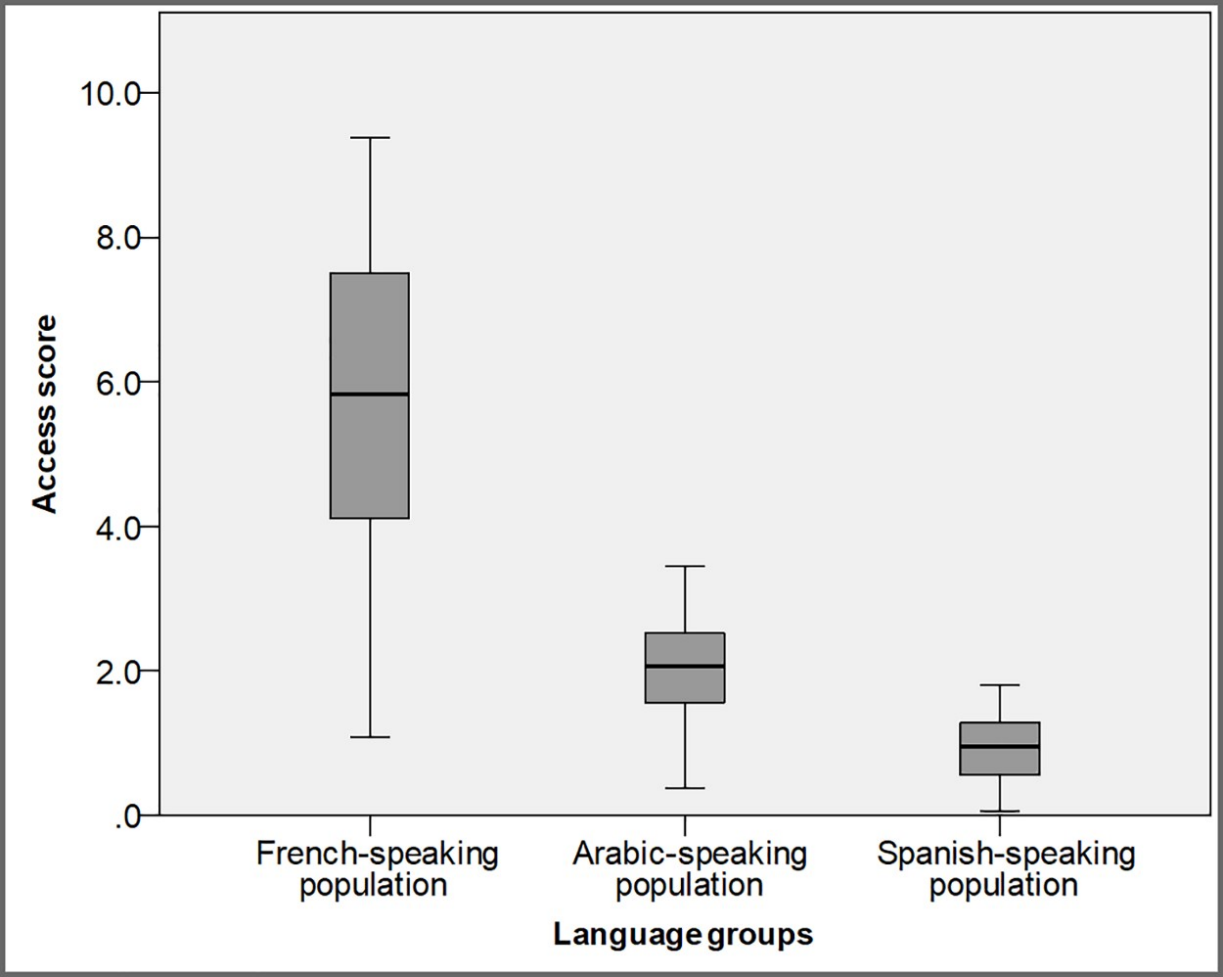
Box-plot showing the distribution of the language-specific geographic accessibility to primary care providers (PCPs) within 15 minutes driving distance.

Minorities, immigrants, and refugees in Canada tend to live in urban areas [27], which are usually associated with better geographic access to health care. However, accessibility is reduced due to barriers accessing language-specific health care. To compare the geographic accessibility measures for minority language speaking PCPs, the access scores were mapped after categorizing into six classes (manually defined classes), where the last two classes indicate DAs with low geographic accessibility (see Fig 3). An additional class (no population) is used to point out DAs having no population that speak a specific language, to differentiate from DAs having no access score. In all cases, the accessibility of each population to PCPs can be identified through the graduated colours, with access score decreasing as the colour gets darker, such that lowest accessibility is found in the DAs with the darkest colour. Further, the language-specific accessibility scores were compared using a Kruskal-Wallis H test (F (2, 1266) = 950.4; p < 0.001) and box-plot (Fig 4).

## Discussion and conclusions

### Interpretation

We estimated the spatial variation in geographic access to primary care services and examined its association with the distribution of vulnerable populations within the city of London, Ontario. In total, there were 267 active PCPs within the city in 2016, which provided 0.77 PCPs per 1000 population (city average). Access scores for all PCPs and PCPs speaking minority languages in the form of PCPs per 1000 population were calculated using E2SFCA method. The focus of this research was to gain a better understanding of how the geographical distribution of primary care services varies at local-scale in a mid-sized, ethnically diverse city in Canada. We sought to demonstrate the importance of spatial aspects of access to PCPs using a GIS-based floating catchment area approach.

The DAs with better geographic accessibility for all PCPs were in the downtown/central and surrounding areas of the city, where the geospatial analysis suggests dissimilarity or mismatch in the distribution of PCPs relative to vulnerable populations. DAs with a high proportion of vulnerable populations versus low access scores situated mainly close to city boundaries particularly for low-income families. In case of seniors and lone parents, high-low can be found along the east side of the city. These findings clearly suggest that some areas have a surplus of PCPs while others have a deficit. This means the high demand needs of vulnerable populations are not being met. This extends earlier findings on the intra-urban disparity of primary care services [13, 28]. This is in accord with other research [for example, 8, 10] which suggests inequalities in the distribution of primary care resources for vulnerable groups in Canadian urban areas. This is important information policy makers and city managers need to know to ensure primary care services are organised around the needs of the population.

The spatial patterns of geographic accessibility scores for PCPs speaking specific minority languages suggest that distribution of PCPs is not matched with the population needs—some areas having a deficit of PCPs providing care in a specific language. In the map of French-speaking PCPs (Fig 3A), overall, the access score is very high (Mean = 2.85, SD = 1.04), but those living close to the city boundaries particularly on the east and south sides have poor accessibility (<1.00) (Fig 4). In the case of access scores for the Arabic-speaking population (Fig 3B), there is very high accessibility (Mean = 1.01, SD = 0.36) with comparatively little variability between the regions of the city (Min = 0.19, Max = 1.73), although the highest accessibility is in the central region and it decreases towards the east and south of London (Fig 4). The accessibility map for the Spanish-speaking population reveals relatively low accessibility levels throughout the city (Mean = 0.47, SD = 0.23), but the lowest accessibility for the Spanish-speaking population is in the south of London (<0.50; Fig 3C).

The geospatial comparative analysis suggests dissimilarity or mismatch in the distribution of PCPs in relation with vulnerable populations. These results further support the idea that geographic accessibility scores for PCPs speaking specific minority languages vary across the city, particularly for the Spanish population, which results in some areas having a deficit of PCPs providing care in a specific (Spanish) language. These findings seem to be consistent with and broadly support the work of other studies which reported that immigrants with different ethnic origins experience language barriers when attempting to access primary care in different Canadian urban areas [9, 11–13, 16, 29, 30]. In addition, the intra-urban disparity in the case of both all PCPs and minority language speaking providers in a mid-sized city resembles what research in large urban centres has shown [10, 13, 14, 30, 31]. For example, using a mixed-methods approach, Wang [14] revealed spatial disparities in access to Tamil-speaking family physicians across the Toronto census metropolitan area.

### Limitation

There are a few limitations that need to be considered when interpreting the results. The E2SFCA method compares the population counts from the 2011 census to 2015 PCPs. While the data were the most recent available at the time of the analysis, using language specific population data from 2011 may result in small inaccuracies particularly in DAs having small population/language specific populations. Similarly, the PCP locations were accurate as of 2015 when the analysis was performed, but as new PCPs open practices and existing PCPs move or retire, level of geographic accessibility will also change. Also, this study uses first language spoken as the dependent variable, however this does not mean that they do not speak English (or French). Since December 2015, there has been a significant influx of newcomers from Syria (Arabic-speaking) in the London area. At the time of this research, the impact of this influx is unknown. One possible question could be regarding the analysis process used to compare the geographic accessibility scores for all PCPs with language specific measures and three vulnerable groups. Indeed, such techniques are not new and are routinely used in geographical research [13, 17, 32, 33].

## Conclusions

The overall access score provides a basic understanding of the status of primary care within the city: there is a variance for each group, as the distribution of PCPs is not matched with the population needs. Even when geographic accessibility is high, there is no guarantee that these PCPs take care of the respective patients. This study provides a geospatial approach that can be used to evaluate the distribution of primary care and other health care services such as mental health services, dental care with respect to population needs across different urban areas within/across provincial jurisdictions. There is a need to develop processes or mechanisms to connect people to the PCP of their choice, or to services and resources in the language of their choice (e.g., French-speaking person to French-speaking PCP, translating online services into different languages). This information may be used to dialogue with the city of London officials and ethno-cultural groups to educate them on gaps that could be addressed through targeted recruitment of health care professionals.

## References

[1] Health Canada. Canada's health care system. Ottawa, ON: Health Canada, 2012.

[2] IPAC (Institute of Public Administration of Canada). Healthcare governance models in Canada: A provincial perspective. Toronto, ON: Institute of Public Administration of Canada, MNP, and Fasken Martineau, 2013 April 8–9, 2013. Report No.

[3] Health Canada. About Primary Health Care Ottawa2006 [cited 2012 16 July]. Available from: http://www.hc-sc.gc.ca/hcs-sss/prim/about-apropos-eng.php#a1.

[4] Franks P, Clancy CM, Nutting PA. Gatekeeping Revisited—Protecting Patients from Overtreatment. New England Journal of Medicine 1992;327(6):424–429. 10.1056/NEJM199208063270613 1625720

[5] Alter DA, Stukel T, Chong A, Henry D. Lesson From Canada’s Universal Care: Socially Disadvantaged Patients Use More Health Services, Still Have Poorer Health. Health Affairs 2011;30(2):274–283. 10.1377/hlthaff.2009.0669 21289349

[6] Clarke J. Difficulty Accessing Health Care Services in Canada Health at a Glance: Statistics Canada; 2016.

[7] Health Council of Canada. Where you live matters—Canadian views on health care quality: results from the 2013 Commonwealth Fund International Health Policy Survey of the General Public. Health Council of Canada, 2014 H173-1/8-2014E-PDF.

[8] Paez A, Mercado RG, Farber S, Morency C, Roorda M. Accessibility to health care facilities in Montreal Island: an application of relative accessibility indicators from the perspective of senior and non-senior residents. International Journal of Health Geographics 2010;9(Journal Article):52–52. 10.1186/1476-072X-9-52 20973969 PMC2987784

[9] Ngwakongnwi E, Hemmelgarn BR, Musto R, Quan H, King-Shier KM. Experiences of French speaking immigrants and non-immigrants accessing health care services in a large Canadian city. International journal of environmental research and public health 2012;9(10):3755–3768. 10.3390/ijerph9103755 23202772 PMC3509478

[10] Bissonnette L, Wilson K, Bell S, Shah T. Neighbourhoods and potential access to health care: The role of spatial and aspatial factors. Health & Place 2012;18(4):841–853. 22503565 10.1016/j.healthplace.2012.03.007

[11] Dastjerdi M. The case of Iranian immigrants in the greater Toronto area: a qualitative study. International Journal for Equity in Health 2012;11(1):9. 22369146 10.1186/1475-9276-11-9 PMC3305531

[12] Lum ID, Swartz RH, Kwan MYW. Accessibility and use of primary healthcare for immigrants living in the Niagara Region. Social Science & Medicine 2016;156:73–79. 27017093 10.1016/j.socscimed.2016.03.024

[13] Wang L. Analysing spatial accessibility to health care: a case study of access by different immigrant groups to primary care physicians in Toronto. Annals of GIS 2011;17(4):237–251.

[14] Wang L. Unequal spatial accessibility of integration-promoting resources and immigrant health: A mixed-methods approach. Applied Geography 2018;92:140–149.

[15] McKeary M, Newbold B. Barriers to Care: The Challenges for Canadian Refugees and their Health Care Providers. Journal of Refugee Studies 2010;23(4):523–545.

[16] Woodgate RL, Busolo DS, Crockett M, Dean RA, Amaladas MR, Plourde PJ. A qualitative study on African immigrant and refugee families’ experiences of accessing primary health care services in Manitoba, Canada: it’s not easy! International Journal for Equity in Health 2017;16(1):5 10.1186/s12939-016-0510-x 28068998 PMC5223444

[17] Dummer TJB. Health geography: supporting public health policy and planning. Canadian Medical Association Journal 2008;178(9):1177–1180. 10.1503/cmaj.071783 18427094 PMC2292766

[18] HealthForceOntario. HealthForceOntario Marketing and Recruitment Agency: HealthForceOntario Marketing and Recruitment Agency; 2018 [cited 2018 May 29]. Available from: http://www.healthforceontario.ca/en/Home.

[19] Schultz SE, Glazier RH. Identification of physicians providing comprehensive primary care in Ontario: a retrospective analysis using linked administrative data. CMAJ Open 2017;5(4):E856–E863. 10.9778/cmajo.20170083 29259018 PMC5741421

[20] Statcan. Census Profile: Canada, provinces, territories, census divisions, census subdivisions and dissemination areas. In: Canada S, editor. Ottawa, ON 2011.

[21] City of London. City of London—Community Profile. London, ON: 2016.

[22] Radke J, Mu L. Spatial Decompositions, Modeling and Mapping Service Regions to Predict Access to Social Programs. Geographic Information Sciences 2000;6(2):105–112.

[23] Luo W, Wang F. Measures of spatial accessibility to health care in a GIS environment: Synthesis and a case study in the Chicago region. Environment and Planning B: Planning and Design 2003;30(6):865–884. 10.1068/b29120 PMC8238135 34188345

[24] Langford M, Higgs G, Fry R. USW-FCA2: An ArcGIS add-In tool to compute Enhanced Two-Step Floating Catchment Area accessibility scores. 2015.

[25] Page N, Langford M, Higgs G. Measuring Spatial Accessibility to Services within Indices of Multiple Deprivation: Implications of Applying an Enhanced two-Step Floating Catchment Area (E2SFCA) Approach. Applied Spatial Analysis and Policy 2017.

[26] Focus on Geography Series, 2016 Census [Internet]. Statistics Canada. 2017 [cited Statistics Canada Catalogue no. 98-404-X2016001]. Available from: https://www12.statcan.gc.ca/census-recensement/2016/as-sa/fogs-spg/Index-eng.cfm.

[27] Statcan. Immigration and ethnocultural diversity in Canada. Ottawa, ON: Statistics Canada, 2011 Contract No.: 99-010-X2011001.

[28] Shah T, Bell S, Wilson K. Spatial Accessibility to Health Care Services: Identifying under-Serviced Neighbourhoods in Canadian Urban Areas. PLOS ONE 2016;11(12):e0168208 10.1371/journal.pone.0168208 27997577 PMC5172578

[29] Asanin J, Wilson K. "I spent nine years looking for a doctor": Exploring access to health care among immigrants in Mississauga, Ontario, Canada. Social Science & Medicine 2008;66(6):1271–1283. 18194831 10.1016/j.socscimed.2007.11.043

[30] Floyd A, Sakellariou D. Healthcare access for refugee women with limited literacy: layers of disadvantage. International Journal for Equity in Health 2017;16:195 10.1186/s12939-017-0694-8 29126420 PMC5681803

[31] Edward J, Biddle DJ. Using Geographic Information Systems (GIS) to Examine Barriers to Healthcare Access for Hispanic and Latino Immigrants in the U.S. South. Journal of Racial and Ethnic Health Disparities 2017;4(2):297–307. 10.1007/s40615-016-0229-9 27129855

[32] Brown EJ, Polsky D, Barbu CM, Seymour JW, Grande D. Racial Disparities In Geographic Access To Primary Care In Philadelphia. Health Affairs 2016;35(8):1374–1381. 10.1377/hlthaff.2015.1612 27503960

[33] Wang F, Luo W. Assessing spatial and nonspatial factors for healthcare access: towards an integrated approach to defining health professional shortage areas. Special section: Geographies of Intellectual Disability 2005;11(2):131–146. 10.1016/j.healthplace.2004.02.003 15629681

